# Multidisciplinary Approach for Full-Mouth Rehabilitation of a Young Adult Patient with Ameloblastoma

**DOI:** 10.1155/2021/8694775

**Published:** 2021-10-19

**Authors:** Hung-Yi Liao, May-Show Chen, Ya-Fen Yang, Pei-Bang Liao, Sheng-Wei Feng, Po-kai Juan

**Affiliations:** ^1^Division of Prosthodontics, Department of Dentistry, Taipei Medical University Hospital, Taipei, Taiwan; ^2^School of Dentistry, College of Oral Medicine, Taipei Medical University, Taipei, Taiwan

## Abstract

Ameloblastoma is a benign but locally invasive neoplasm of the odontogenic epithelium that tends to grow slowly in the mandible or maxilla. It can be highly destructive to the surrounding dental anatomy and can cause death by progressive spread to nearby vital structures in rare cases. Marginal resection is the most effective method of eliminating the tumor, but treatment can further contribute to oral and dental deformity and malfunction. This clinical report describes the dental rehabilitation of a young adult patient diagnosed with ameloblastoma and underwent preliminary marsupialization, segmental mandibulectomy, and fibula free flap reconstruction, followed by mandibular dental implant placements. Orthodontic and rapid palatal expansion for maxillary arch correction was also performed. The treatment goal of regaining dental function and a satisfactory appearance was accomplished.

## 1. Introduction

Ameloblastoma is a benign odontogenic tumor that arises in the jawbone. This tumor occurs in three different variants: multicystic, unicystic, and peripheral, which deserve separate consideration because of differing therapeutic considerations and prognosis [1]. Overall, the tumor is slow growing and locally invasive and has a high recurrence rate. Marsupialization is useful as a preliminary treatment of cystic ameloblastoma.

However, following marsupialization, the tumor still has the potential to infiltrate into the surrounding tissues [2]. Therefore, marginal resection with a safety margin is considered the most effective method of eliminating the tumor. However, it also causes facial deformation and dysfunction, which leads to a decrease in a patient's living quality.

A multidisciplinary approach is needed for the patient's complete rehabilitation, including bone grafting, implant placement, and prosthetic fabrication. Sometimes treatment must combine orthodontic treatment to create an optimum occlusal relationship and gain sufficient space for ideal reconstruction. This report presents the long-term clinical follow-up of a patient with ameloblastoma who successively underwent surgical, orthodontic, and prosthetic treatment for complete oral reconstruction.

## 2. Case Report

**Figure 1:**
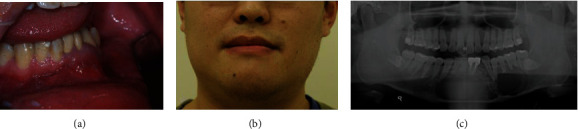
Initial findings. (a) Intraoral view shows a raised mass over the left buccal vestibular area. (b) Extraoral view shows slight facial asymmetry. (c) Panoramic radiograph reveals multilocular radiolucent lesion over the anterior and left mandible.

A 26-year-old man came to the Oral and Maxillofacial Surgery Department of Taipei Medical University Hospital (Taipei, Taiwan) in May 2011 due to swelling and pain in the jaw. The intraoral examination revealed a raised mass in the left mandibular buccal vestibular area extending to the labial area without secretory discharge. The color and appearance of the surrounding mucosa were normal. The teeth in this area also have no obvious periodontal pockets (Figure 1(a)). On extraoral examination, the patient's lateral view shows a protruding mandible, and the left and right sides of the face were also slightly asymmetrical. The neurological examination was normal, and there were no swollen and palpable lymph nodes in the neck (Figure 1(b)). A radiographic examination showed a wide range of radiolucent lesions in the mandible from the canine area on the right to the second molar on the left. The biopsy and pathology reports confirmed that the lesion was consistent with ameloblastoma (Figure 1(c)). The pathology report is presented as follows. Grossly, they are gray and brown and elastic with some bone chips. Microscopically, it shows an ameloblastoma with islands of odontogenic epithelium in plexiform and follicular patterns within a fibrous stroma. The odontogenic epithelium shows peripheral palisading and stellate reticulum. Neither cytological atypia nor increased mitoses is seen.

**Figure 2:**
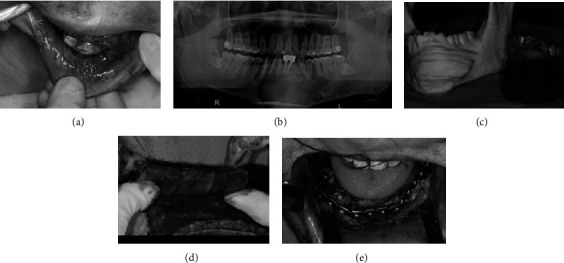
Mandible reconstruction. (a) A soft liner was used as the obturator material after two marsupializations. (b) The tumor size decreased on the left side but remained unchanged in the anterior area. (c) The 3D model was used to simulate the scope of the resection. (d) The free fibular flap was positioned. (e) The flap was fixed with the prebent metal bone band.

Marsupialization was performed to reduce the size and destructiveness of the tumor for subsequent resection surgery. The marsupialization was firstly performed in the anterior and left posterior region of the mandible. Soft liner was applied immediately as the obturator materials, and regular visits were arranged to monitor the changes of tumor size and to adjust the obturator (Figure 2(a)). After 4 months, the tumor size was gradually reduced on the left posterior region, but remained unchanged over the anterior region. Thus, the marsupialization was performed again over the anterior region. It took a total of 12 months to reduce the size of the lesion and confirm the scope of the tumor (Figure 2(b)). After that, the segmental mandibulectomy combined with fibula free flap reconstruction was conducted. At that time, three-dimensional (3D) image processing and printing were used to analyze and guide dental implant design and construction. The more precise the reconstruction operation can be, the more the damage can be reduced. Additionally, the entire postoperative period will benefit from precision reconstruction.

Therefore, computed tomography (CT) examination was taken to evaluate the precise extension and region of the tumor. And then, the 3D printing transferred from CT files/images was applied to create a solid model, and the actual scope of the resection could be simulated before surgery (Figure 2(c)). A fibula 3D model was also exported for model surgery, surgical template making, and titanium bone plate bending. During the surgery, the defect was first fixed with a prebent metal bone plate, and then, the fibula part of the removed free flap was divided into three sections according to the surgical template and bent to meet the needs of the defect. Soft tissue defects in the mouth were also reconstructed with free flaps (Figures 2(d) and 2(e)). With the 3D printed models, the surgery can be completed more accurately, and the treatment time can be significantly reduced.

**Figure 3:**
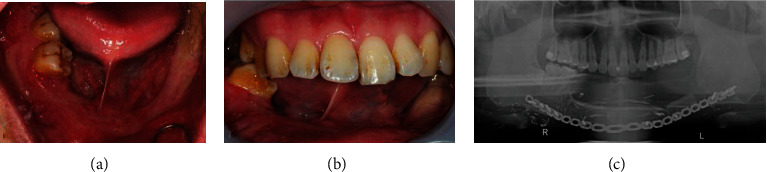
The soft and hard tissues had healed completely and ready for reconstruction. (a, b) Intraoral view. Teeth 46 and 47 were temporarily kept for maintaining vertical support. (c) Panoramic radiograph after segmental mandibulectomy and fibular free flap.

During the healing period, the patient could only use the remaining teeth, 46 and 47, and the opposite tooth. After 9 months, alveoloplasty was performed to correct the uneven and sharp bony edges. The patient was referred to a prosthodontist 3 years after the first visit. At this point, the soft and hard tissues had healed completely and were ready for reconstruction (Figure 3).

After a prosthetic evaluation, the problems and preliminary treatment directions were revealed. The patient's treatment program was developed to address the following issues. Full mandible implants were necessary and feasible to meet the physical and psychological needs of the patient. Considering that teeth 46 and 47 were the only two teeth that could be used for chewing, a staged dental implant approach was adopted. The transverse discrepancy in the upper and lower jaws and a deficiency in the right maxilla resulting in asymmetric facial features required resolution but could not be repaired by dentures alone. The cross-bite on the right side required resolution, and the space was limited for restoration on the right side.

**Figure 4:**
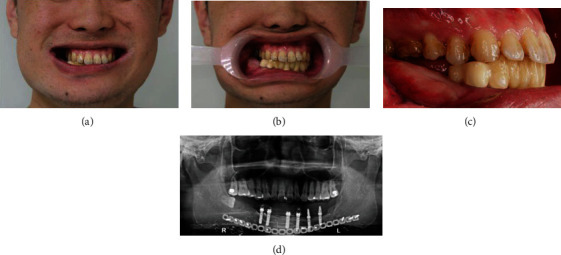
Teeth 46 and 47 were extracted after staged approach implant placement. Cross-bite and lack of restoration space over right side were observed. (a, b) Extraoral view shows narrow maxillary arch and large buccal corridor. (c) Lack of restoration space over lower right posterior area. (d) Panoramic film after staged approach implant placement.

**Figure 5:**
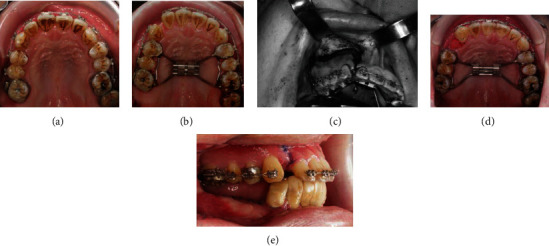
(a) Leveling and alignment after extraction of tooth 17 to create space. (b) Placement of the rapid palatal expander. (c) Posterior segmental osteotomy in the right maxilla. (d) Complete maxillary arch expansion. (e) Correction of the transverse jaw deficiency, with increased restoration space.

**Figure 6:**
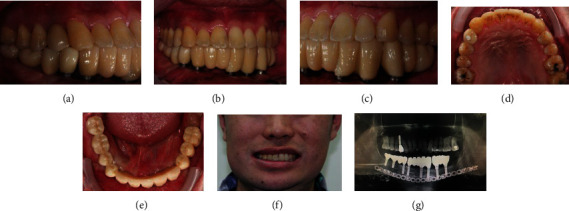
Completed dental reconstruction. (a–c) Final insertion of zirconia implant-supported fixed partial dentures and ideal occlusal plane and occlusion. (d, e) Smooth and symmetrical dental arches. (f) Photograph showing improved asymmetry of face. (g) Radiograph of the final mandible and dental reconstruction.

A staged approach was conducted. First, four implants in tooth 33, 32, 43, and 44 position were placed in the mandible. After the osseointegration, implant-supported provisional crowns 3332xxx4344 were installed in position. Then, two implants in teeth 34 and 36 were placed on the left mandible and were fitted with 34 × 36 provisional crowns. Finally, teeth 46 and 47 were extracted (Figure 4). The purpose of the patient's orthodontic treatment was to gain restoration space on the right side and correct the upper jaw deficiency. In the beginning, tooth 17 was extracted and followed by leveling and alignment to create space (Figure 5(a)). After rapid palatal expander (RPE) placement (Figure 5(b)), the right maxillary posterior segmental osteotomy (PSO) (Figure 5(c)), right mandibular alveoloplasty, and extraction of tooth 48 were performed in the same operation to achieve proper interocclusal relationship and gain enough restoration space over right posterior region. Next, the patient was asked to regularly rotate the screw of the conventional Hyrax expander. One rotation per day produces a 0.2 mm lateral expansion, which lasts up to two weeks but varies from individual to individual. After the desired position was achieved, there was no need to continue turning the screw. However, it was still necessary to continue wearing the device for at least three months to stabilize the expansion. PSO and RPE provided the required lateral expansion effect to transform the upper arch into a better shape. Alveoloplasty also increased restoration space (Figures 5(d) and 5(e)). The implant surgery was completed with the implantation of teeth 14, 46, and 47. The provisional teeth were installed after osseointegration. The lower jaw was divided into three groups of cement-retained bridges, and tooth 14 was a screw-retained crown. The prosthesis was made with zirconia-based material. The pontic type was designed to be consistent with the original provisional crowns and appropriate for the patient's extensive mandibular reconstruction. This design did not allow any contact between the pontic bottom and the gums so that the patient could clean the teeth as efficiently as before, and the large-scale bottom hollow was not prone to food impaction. After postoperative treatment was completed, the upper and lower jaws presented smooth and symmetrical dental arches, dental function was restored, and the patient's face showed improved symmetry (Figure 6).

## 3. Discussion

Ameloblastoma is an odontogenic tumor with highly aggressive and curable features. Nonetheless, a consensus has not been reached on the biologic behavior of this neoplasm and how best to treat it [3]. Because of its highly infiltrative, aggressive nature and to reduce the risk of recurrence, resection of the tumor with 1.0-2.0 cm linear bony margins followed by free autologous grafting is recommended [4]. Hidalgo reported the utility of vascularized fibula flaps for mandibular reconstruction in 1989 [5]. Since then, it has become the first option for mandible reconstruction [6, 7]. A significant advantage is that fibular free flap reconstruction offers adequate bony dimensions and bone quality, allowing for the placement and osseointegration of dental implants and the possibility of restoring masticatory function and aesthetics [8]. The flap operation is difficult to control during conventional surgery and occasionally results in dissatisfying occlusion and appearance. Virtual planning and 3D print modeling using preoperative computed tomographic (CT) data have recently been introduced. The true-to-size models and templates are easy to obtain, and prebending of a titanium plate can be done on the model. These techniques permit more accurate and time-saving reconstruction [9].

We encountered several problems throughout the postoperative prosthetic evaluation, including transverse maxillomandibular discrepancy and right maxillary arch deficiency. To correct the malocclusion and make the buccal corridor less obvious, we applied an RPE for orthodontic treatment appropriate for the patient's age and facial structure. Rapid palatal expansion is a technique whereby expansion of 0.5 mm to 1 mm is achieved each day until the posterior cross-bite is relieved. For stability purposes, the RPE usually remains in the patient's mouth for 3–6 months, but this time may vary among patients [10]. In our patient, implant placement was performed via a staged approach for maintaining oral function [11, 12]. The number of implants was increased as much as possible to avoid inappropriate prosthetic design due to a small number of implants.

## 4. Conclusion

This clinical report illustrated an interdisciplinary treatment approach provided by cooperation between maxillofacial surgeons, prosthodontists, and orthodontists. After eight years of treatment, the patient had no evidence of tumor recurrence, completely reconstructed soft and hard tissues, restored function, improved appearance, and ultimately quality of life. Reconstruction after facial tumor resection remains a challenge. In our patient, a rigorous treatment plan, coupled with a multidisciplinary approach, achieved the treatment goal of restoring the patient's oral and dental function and appearance.

## Data Availability

The data during the current study are available from the corresponding author on a reasonable request.
